# Post-Infection Symptoms in U.S. Soldiers with Malaria During the Second World War: Major Limitation to Return to Duty

**Published:** 2026-03-06

**Authors:** G. Dennis Shanks

**Affiliations:** Australian Defence Force Infectious Disease and Malaria Institute, Enoggera, Queensland, Australia; School of Public Health, University of Queensland, Brisbane, Australia

## Abstract

Malaria proved decisive in determining the outcome of the Pacific theater during the Second World War. In 1943 alone, over 100,000 malaria cases were reported among the U.S. military in the Southwest Pacific and South Pacific. Thousands of sick soldiers were evacuated from their units and hospitalized for weeks or months of rehabilitation due to malaria. The primary challenge was not treatment of acute infections, as death rates were very low, but rather an inability to return recovered soldiers quickly to their units. Relapsing *Plasmodium vivax* malaria posed a particular problem, with many soldiers stationed at Guadalcanal or New Guinea suffering more than 10 relapses. Secondary gain from residual symptoms became apparent when around 1% of malaria patients were repatriated for ‘chronic malaria’. Future conflicts disrupted by infectious diseases will almost certainly include diffuse, post-infection symptoms that must be anticipated to prevent catastrophic war-fighter attrition.

*“However, the way the individual adjusted to the malaria and concurrent situational factors, contributed to the development of symptoms, to their perpetuation and intensification.” ^1^*

Nearly all U.S. soldiers deployed to the Pacific theater during the Second World War were hospitalized at least once per year, ^2^ primarily for infectious diseases—such as malaria, scrub typhus, filariasis, and skin infections—rather than combat wounds. ^3^ Malaria came close to being a decisive agent in the Pacific theater due to the sheer number of casualties it produced. Infection rates reached 250 per 1,000 men per year in the Solomon Islands and New Guinea. ^4^ **Figure 1** shows the variety of hospitalizations in Guadalcanal, in the Solomon Islands, in 1942-1943, with a majority due to malaria. ^3^ At the end of 1942, entire units had been incapacitated by malaria in Milne Bay, New Guinea due to inadequate chemoprophylaxis and preventive measures, but fortunately after the combined Australian and U.S. forces had already defeated the Japanese invasion the previous August. ^5^

**FIGURE 1.**
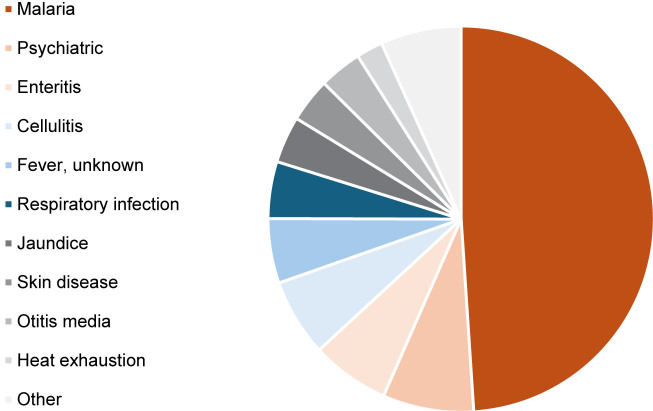
Disease Casualties at Three Provisional Field Hospitals Demonstrating Malaria Predominance, U.S. Army 101st Medical Regiment, Americal (23rd) Infantry Division, Guadalcanal, November 1942–February 1943 ^3^

From the viewpoint of military commanders, the most critical limitation of malaria infection was that infantry divisions withdrawn from the Solomon Islands or New Guinea became useless for further deployment for at least 6 months. ^4^ Multiple relapses of malaria struck soldiers even while their divisions attempted to reconstitute in non-endemic areas such as Australia and Fiji. Military planners estimated that maintaining contact with the enemy by 1 division required at least 3, possibly as many as 5, divisions simply because of malaria casualties. ^5 , 6^ Relapsing malaria due to *Plasmodium vivax* was a common sequela that often led to multiple, sequential febrile attacks even when a soldier was removed from an endemic area. ^7^

Two weeks of anti-malarial drug treatment was required for those sick enough to be hospitalized. Many soldiers were medically evacuated from combat zones due to limited medical support in forward areas. ^8^ By 1943, the situation had become unsustainable. Eventually, improved regimens of enforced chemo-suppression with quinacrine, combined with better anti-mosquito measures, reduced new infections, and treatment regimens were shortened to 7 days. Use of 8-aminoquinolines to eliminate latent parasites causing relapse would have to wait for chemotherapeutic advances during the Korean War, however. ^9^

While treatment with quinine and quinacrine (atabrine) proved successful, and death rates remained very low, ^6^ a more insidious problem for the U.S. military emerged. Large numbers of soldiers developed chronic symptoms and weight loss that led to repatriation for ‘chronic malaria’. Chronic malaria was characterized not only by multiple relapses—10 were not unusual—but a failure to recover between nearly monthly febrile relapses. Soldiers suffering from chronic malaria populated a medical system designed to treat combat injuries, with 3,334 malaria evacuations to the U.S. from the South Pacific in 1943, and a similar number from the Southwest Pacific to Australia. ^3 , 8^

The magnitude of the problem prompted the U.S. military to designate entire Army general hospitals as specialty centers for tropical diseases: in Longview, Texas; Modesto, California; Swannanoa, North Carolina; and in Klamath Falls, Oregon, for the U.S. Navy and Marine Corps; in addition to the 105th General Hospital in Gatton, Australia. ^4^ Those dedicated facilities were clearly preferrable to the tented field hospitals **(Figure 2)** . The farther a malaria-infected soldier traveled from where he acquired infection, the better the treatment facilities became—and more removed the opportunity to return to his original unit. Secondary gains from continued symptoms increased proportionally.

**FIGURE 2.**
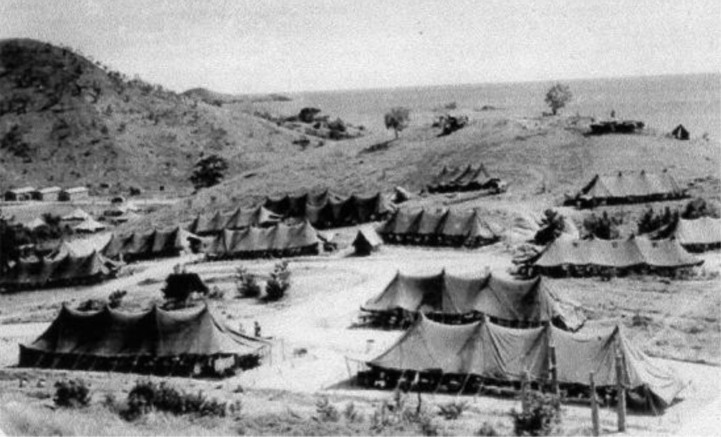
U.S. Army Hospital, Advanced Base Near Port Moresby, Papua New Guinea, August 1943 ^16^

The concern over chronic malaria grew so severe that medical studies were initiated in both Australia and Fiji to determine better ways to limit disease casualties. After studying 3,358 malaria patients in Australia in 1943-1944, officials found a wide range of responses to malaria infection among service members. ^8^ Many soldiers reported chronic weakness and a variety of ill-defined complaints including headaches, dizziness, nervousness, insomnia and tremor. In Fiji, largely working with soldiers from the Americal (23rd) Infantry Division, a group of psychiatrists conducted a medical and laboratory study of malaria groups at the 18th General Hospital, ^1^ and found similarly wide variation in soldiers' abilities to deal with malaria infection. Those who tolerated the disease poorly primarily reported weakness and chronic fatigue, along with a host of ancillary complaints. Remarkably, the only definite physical finding from the studies of malaria casualties was that most soldiers had lost 10-20 pounds of body weight since developing malaria.

The results of those wartime studies concluded that the non-physical effects of malaria were largely psychosomatic in nature. It was ultimately determined that patients—and the U.S. Army—achieved better outcomes when chronic malaria's psychosomatic element was recognized and its medicalization was minimized. ^8^ One wartime study author observed, “The soldier is usually capable of remaining useful, even though sometimes in a limited capacity, so long as his morale remains satisfactory; and symptomatology only becomes severe when the adjustment of the person is faulty.” ^1^ Although malaria infection was nearly universal for frontline infantry, the vast majority of soldiers coped well with the stress and only required hospitalization when overcome by 40° Celsius fevers and uncontrollable rigors.

Neuropsychiatric casualties due to maladjustment were not new in the Pacific theatre. All humans have limitations on abilities to cope with stress, and soldiers in the Pacific theater found themselves in life-threatening situations in a tropical jungle, with malaria an added stress in an austere warfare environment. Soldiers whose coping mechanisms failed early showed up as combat stress casualties. In mid-1943, after landing on New Georgia in the Solomon Islands, the 43rd Division had been incapacitated by war neurosis and combat stress resulting in 16% medical evacuations. ^10^ Fully 15% of medical evacuations from the South Pacific in 1943 were due to neuropsychiatric diagnoses, with likely considerable overlap with other diseases such as malaria. ^3^

Chronic malaria manifested later in the World War II Pacific conflict, when soldiers consciously or unconsciously understood that illness would keep them from returning to a combat zone. Medicalizing the symptoms of either combat stress or malaria was counter-productive and likely extended soldier hospitalizations during the war. The treatment of combat stress casualties was subsequently developed with emphasis on proximity, immediacy, and expectancy—principles that greatly influenced recommendations for handling post-infection casualties. Malaria treatment units were created near the front lines and evacuation distances were minimized. These strategies conformed to the principles of combat stress treatment and succeeded even when the U.S. Army encountered drug-resistant malaria during the Vietnam War, proving highly effective for management of post-infection casualties. ^11^

While malaria is unlikely to recur as a major casualty-producing agent in current South China Sea scenarios, the recent COVID-19 pandemic demonstrated both our limited ability to predict future epidemics and the potency of chronic disabling conditions such as the poorly defined ‘long COVID’. ^12^ Current INDOPACOM (Indo-Pacific Command) military exercises can expose service members to scrub typhus, also likely to have post-infection symptoms, given its potential for cardiovascular damage. ^13^ Most infectious diseases have post-infection symptoms, seen during World War II, with filariasis, and during the Vietnam conflict, with dengue infections. ^14 , 15^

Given the ability of disinformation to spread via the internet, along with the expectation of many soldiers that infections will cause chronic symptoms, future military medical officers will almost certainly find themselves in situations analogous to those in the South Pacific in 1943. Applying the same treatment principles established for combat stress neuropsychiatry—namely proximity, immediacy, and expectancy—for infectious diseases is likely to be successful in minimizing preventable casualties during any future conflict.

## References

[1] Tumulty PA , Nichols E , Singewald ML , Lidz T . An investigation of the effects of recurrent malaria: an organic and psychological analysis of 50 soldiers . Medicine (Baltimore) . 1946 ; 25 : 17 - 75 . doi: 10.1097/00005792-194602000-00002 21014709

[2] Thomas HM . Southwest Pacific area. In: Medical Dept., U.S. Army ; Coates JB , ed. Internal Medicine in World War II , Volume I : Activities of Medical Consultants. Office of the Surgeon General, Dept. of the Army ; 1961 : 473 - 568 . Accessed Feb. 2, 2026 . https://apps.dtic.mil/sti/tr/pdf/ada286772.pdf

[3] Baker BM . South Pacific area. In: Medical Dept., U.S. Army ; Coates JB , ed. Internal Medicine in World War II , Volume I : Activities of Medical Consultants. Office of the Surgeon General, Dept. of the Army ; 1961 : 569 - 623 . Accessed Feb. 2, 2026 . https://apps.dtic.mil/sti/tr/pdf/ADA286772.pdf

[4] Hoff EC. Malaria . In: Medical Dept., U.S. Army ; Coates JB , ed. Preventive Medicine in World War II , Volume VI : Communicable Diseases. Office of the Surgeon General, Dept. of the Army ; 1963 .

[5] Walker AS . The Island Campaigns (Volume III) . In: Australia in the War of 1939–1945, Series Five: Medical . Australian War Memorial ; 1957 . Accessed Feb. 2, 2026 . https://www.awm.gov.au/collection/C1417326

[6] Joy RJ . Malaria in American troops in the south and southwest Pacific in World War II . Med Hist . 1999 ; 43 : 192 - 207 . doi: 10.1017/s002572730006508x 10885139 PMC1044732

[7] Shanks GD . *Plasmodium vivax* relapse rates in Allied soldiers during the Second World War: importance of hypnozoite burden . Am J Trop Med Hyg . 2022 ; 107 : 1173 - 1177 . doi: 10.4269/ajtmh.22-0546 36343595 PMC9768259

[8] Levine HD . Medical experiences with American troops in the Pacific: with remarks on the diagnostic value of sternal puncture in malaria and on the innocuousness of hookworm infection . NEJM . 1946 ; 235 : 933 - 938 . doi: 10.1056/nejm194612262352604 20277658

[9] Zottig VE , Shanks GD . Historical perspective: the evolution of post-exposure prophylaxis for *vivax* malaria since the Korean War . MSMR . 2021 ; 28 ( 2 ): 8 - 10 . Accessed Feb. 2, 2026 . https://www.health.mil/reference-center/reports/2021/02/01/medical-surveillance-monthly-report-volume-28-number-02 33636086

[10] Hallam FT . War neuroses. Appendix G . In: Mullins W , ed. Neuropsychiatry in World War II , Volume II : Overseas Theaters. Office of the Surgeon General, Dept. of the Army ; 1973 : 1063 - 1069 . Accessed Feb. 2, 2026 . https://collections.nlm.nih.gov/catalog/nlm:nlmuid-0211560X2-mvpart

[11] Modell W . Malaria and victory in Vietnam: the first battle against-drug-resistant malignant malaria is described . Science . 1968 ; 162 : 1346 - 1352 . doi: 10.1126/science.162.3860.1346 4880851

[12] Hitchcock S , Cintron SA , Kasuske L , Diaz FJ , Pierce J . Post-COVID-19 condition in military personnel . Mil Med . 2024 ; 189 : e1277 - e1281 . doi: 10.1093/milmed/usad453 38197253

[13] Suhr R , Belonogoff S , McCallum F , Smith J , Shanks GD . Scrub typhus outbreak among soldiers in coastal training area, Australia, 2022 . Emerg Infect Dis . 2024 ; 30 : 41 - 46 . doi: 10.3201/eid3014.240056 39530857 PMC11559576

[14] Russell PK , Ognibene AJ . Dengue and dengue shock syndrome . In: Ognibene AJ , ed. Internal Medicine in Vietnam, Volume II: General Medicine and Infectious Diseases . Office of the Surgeon General, Dept. of the Army ; 1982 : 91 - 98 . Accessed Feb. 2, 2026 . https://achh.army.mil/history/book-vietnam-genmedvn-ch05

[15] Shanks GD , Smith JK . Lymphatic filariasis in soldiers exposed in INDOPACOM . MSMR . 2024 ; 31 ( 8 ): 20 - 23 . Accessed Feb. 2, 2026 . https://www.health.mil/news/articles/2024/08/01/msmr-filariasis-indopacom PMC11413130 39255521

[16] WW2 Military Hospitals: Pacific Theater of Operations and Minor Theaters . WW2 Medical Research Centre . 2024 . Accessed Feb. 2, 2026 . https://www.med-dept.com/articles/ww2-military-hospitals-pacific-theater-of-operations

